# Selectivity in Tip-Induced Skeletal Editing via Heteroatom Substitution

**DOI:** 10.1021/jacs.6c12567

**Published:** 2026-09-24

**Authors:** Shantanu Mishra, Rasmus Svensson, Valentina Malave, Florian Albrecht, Manuel Vilas-Varela, Henrik Grönbeck, Leo Gross, Diego Peña

**Affiliations:** † Department of Physics and Astronomy, 11248 Chalmers University of Technology, 412 96 Göteborg, Sweden; ‡ Competence Centre for Catalysis, 11248 Chalmers University of Technology, 412 96 Göteborg, Sweden; § Center for Research in Biological Chemistry and Molecular Materials and Department of Organic Chemistry, 16780 University of Santiago de Compostela, 15782 Santiago de Compostela, Spain; ∥ 54174 IBM Research, 8803 Rüschlikon, Switzerland; ⊥ Oportunius, Galician Innovation Agency, 15702 Santiago de Compostela, Spain

## Abstract

Skeletal editing enables precise structural modifications of molecules at late stages of a synthetic sequence. We recently demonstrated skeletal editing on a single-molecule scale. Voltage pulses applied by the tip of a scanning probe microscope to an oxygen-containing seven-membered heterocycle led to both oxygen deletion and ring-contraction rearrangement reactions. An open question is whether selective skeletal editing of a heterocyclic core can be achieved by the choice of heteroatom. Here we show that tip-induced reactions of an analogous sulfur-containing seven-membered ring mainly result in sulfur deletion. Our results demonstrate that the combination of tip-induced chemistry and heteroatom selection in the molecular design is a powerful strategy for single-molecule skeletal editing, potentially enabling diverse structural transformations of heterocyclic frameworks.

## Graphical abstract

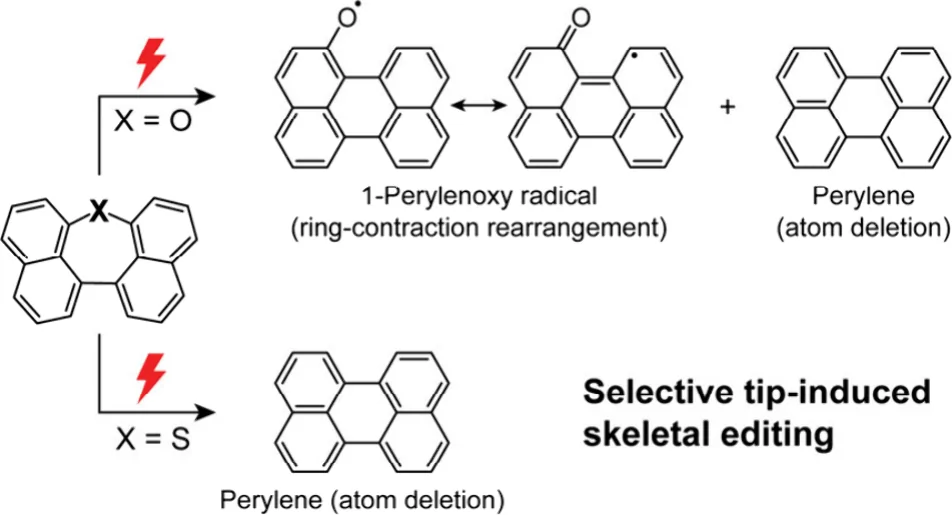

Skeletal editing refers to the late-stage modification of a molecular skeleton through insertion, deletion, or exchange of individual atoms (Scheme 1a). The field has received considerable attention as a means of reducing the need for *de novo* synthesis, and precise structural transformation of molecules,^1,2^ with potential applications in drug discovery, green chemistry, and materials science.^3–5^ In particular, deletion of a strategically placed heteroatom from a carbon-based cyclic skeleton can provide access to diverse carbocyclic motifs. While conventionally performed via solution-phase chemistry (with demonstrated examples of boron,^6^ nitrogen,^7–10^ oxygen,^11,12^ and sulfur^13^ deletion), extension of atom deletion to the single-molecule scale via tip-induced chemistry^14,15^ could enable the synthesis of elusive molecules. In pursuit of single-molecule skeletal editing, we recently explored tip-induced reactions of individual dinaphtho­[1,8-*bc*:1′,8′-*ef*]­oxepine (**DNO**) molecules adsorbed on NaCl (Scheme 1b).^16^ Voltage pulses applied by the tip of a combined scanning tunneling microscope (STM) and atomic force microscope (AFM) to individual **DNO** molecules led to multiple reactions. Among all reacted molecules, nearly 50% corresponded to 1-perylenoxy radical resulting from ring-contraction rearrangement, whereas 33% corresponded to molecules generated via oxygen deletion, of which perylene was the major species (14% of all reacted molecules). The remaining molecules resulted from undesired side reactions. An open question is to what extent the selectivity of skeletal editing in a heterocyclic core can be directed toward a specific reaction by the choice of the heteroatom. In the present work we explore if heteroatom deletion can be favored over ring-contraction rearrangement by replacing oxygen with sulfur, hypothesizing that the lower C–S bond dissociation energy relative to the C–O bond would promote heteroatom deletion. We show that tip-induced reactions of dinaphtho­[1,8-*bc*:1′,8′-*ef*]­thiepine (**DNT**), which shares the same molecular structure as **DNO** but with sulfur replacing the oxygen atom, result in heteroatom deletion in virtually all cases, with perylene as the predominant product (Scheme 1c).

**Scheme 1:**
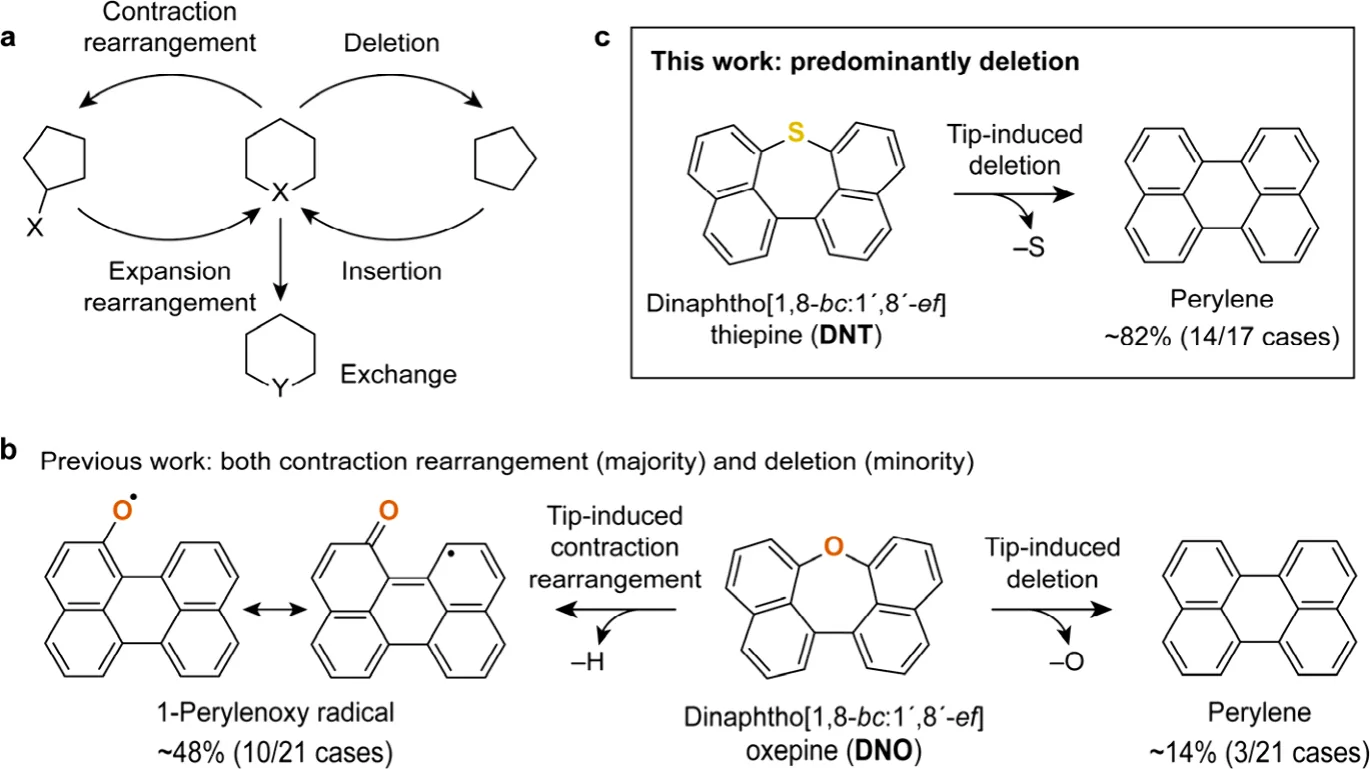
(a) Illustration of Skeletal Editing;^a^ (b, c) Schemes Showing the Major Products Formed from Tip-Induced Skeletal Editing of (b) DNO and (c) DNT

a X and Y denote individual atoms.

**DNT** was synthesized via solution-phase chemistry (Scheme S1 and Figures S1 and S2) and deposited onto a single-crystal Cu(111) surface partially covered by two monolayer-thick (denoted bilayer) NaCl films (Figure S3). Similar to **DNO**, individual **DNT** molecules adsorbed on both Cu(111) and NaCl adopt a geometry where the sulfur atom points toward the surface. Figure 1a–c shows STM and AFM images of a **DNT** molecule adsorbed on Cu(111). Related to the nonplanar geometry of **DNT**, two diagonally opposite benzenoid rings appear bright in AFM imaging due to their upward tilt. The remaining two benzenoid rings, which exhibit a downward tilt, appear notably dark, while the sulfur atom is not visible. Figure 1d–f shows STM and AFM images of a **DNT** molecule adsorbed on NaCl, which exhibits stronger nonplanarity because of weaker molecule–surface interaction on NaCl compared to Cu(111) (see also Figure S4). The streaks in AFM images of **DNT**/NaCl (highlighted in Figure 1e) likely result from conformational switching of the molecule due to interaction with the tip. The adsorption geometry inferred from AFM imaging is in line with the density functional theory (DFT) calculation of the adsorption geometry of **DNT** (Figure 1g,h). Further measurements on **DNT** are shown in Figures S5 and S6.

**Figure 1:**
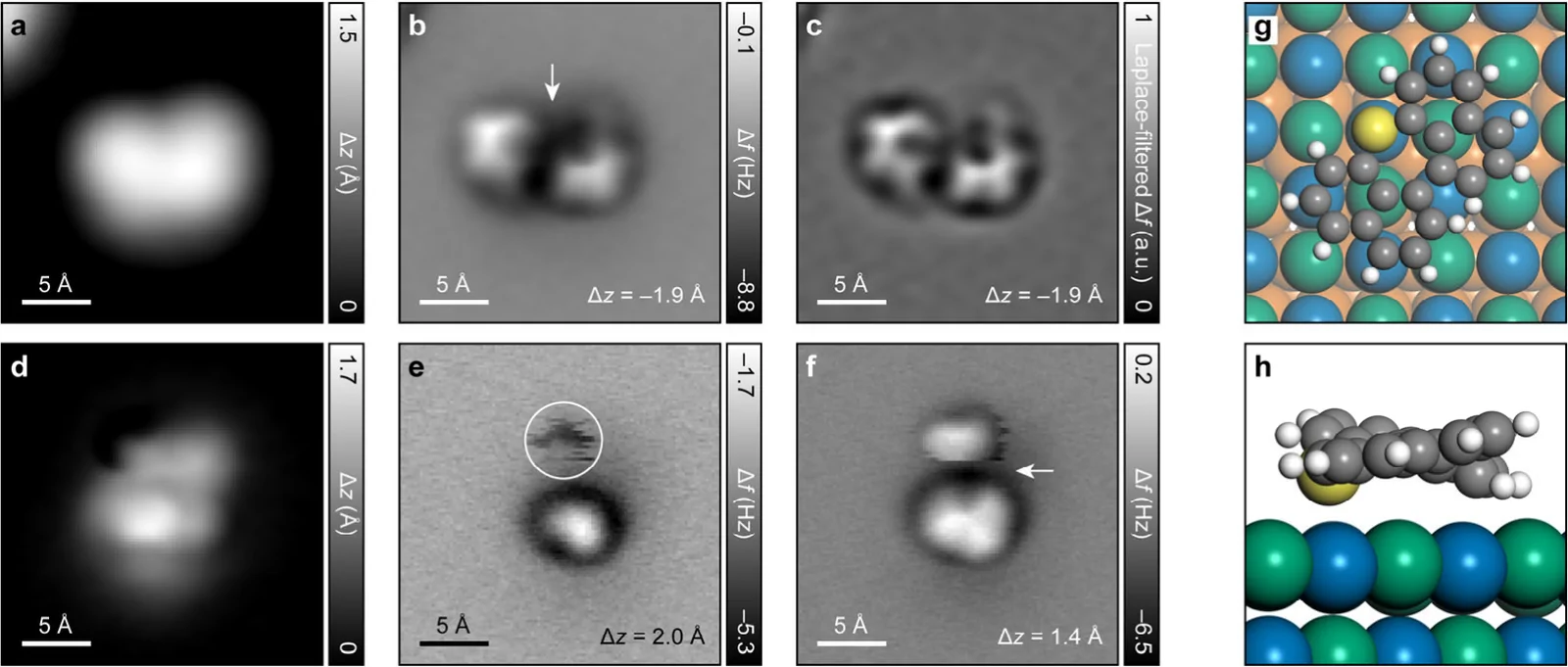
STM and AFM imaging of **DNT**. (a) STM image of a **DNT** molecule adsorbed on Cu(111). (b) AFM image and (c) the corresponding Laplace-filtered image of the **DNT** molecule in (a). Δ*z* denotes the tip-height offset, with positive (negative) values indicating tip retraction (approach) from the set point; Δ*f* denotes the frequency shift; and a.u. denotes arbitrary units. (d) STM image of a **DNT** molecule adsorbed on bilayer NaCl. (e, f) AFM images of the **DNT** molecule in (d) taken at two different tip heights. The circle in (e) highlights streaks likely resulting from conformational switching of the molecule. The arrows in (b) and (f) indicate the locations of the sulfur atom. Scanning parameters for STM images: *V* = 0.2 V, and *I* = 0.30 pA (a) and *I* = 0.25 pA (d). STM set-point for AFM images: *V* = 0.2 V, and *I* = 0.50 pA on Cu(111) (b, c) and *I* = 0.25 pA on bilayer NaCl (e, f). All images were acquired with the same tip. (g) Top and (h) side views of the DFT-calculated adsorption geometry of **DNT** on bilayer NaCl/Cu(111). Atomic color codes: H (white), C (gray), Na (blue), S (yellow), Cl (green) and Cu (brown).

We applied voltage pulses to individual **DNT** molecules adsorbed on NaCl to trigger skeletal editing reactions (we did not observe skeletal editing on Cu(111), see Figure S7). Briefly, we first positioned the tip above a **DNT** molecule at set-point conditions of *V* = 0.2 V and *I* = 0.5 pA (*V* and *I* denote bias voltage and tunneling current, respectively). Then, we opened the feedback loop and retracted the tip by 9.5–12.0 Å to limit the current (typically, *I* < 30 pA at the final voltage). Finally, we increased the voltage to 4.7–5.0 V for 140 ms. If subsequent STM imaging indicated a change in the appearance of the molecule (Figure S8), we conducted AFM imaging to obtain the molecular structure. Upon application of voltage pulses to molecules adsorbed on a defect-free region on bilayer NaCl, we never found those molecules in a 200 Å × 200 Å scan frame. We presume that the molecules were picked up by the tip or displaced from NaCl islands onto Cu(111). Therefore, we applied voltage pulses to molecules adsorbed on bilayer NaCl at the step edges of third-layer NaCl islands, where the adsorption is stabilized. From 47 voltage pulses applied to 28 **DNT** molecules, we observed 17 molecules that underwent a reaction on the surface. In unsuccessful cases, the molecules were either displaced on NaCl without any reaction or not found. Sulfur deletion was the predominant reaction in successful cases (Figure 2), accounting for 16/17 reacted molecules (94%) and resulting in the generation of perylene (14/17 cases, ∼82%) and, in a minority of cases, the perylenyl radical (compound **1**, 2/17 cases). In one case, we observed the generation of compound **2** resulting from a side reaction. Notably, there was no ring-contraction rearrangement observed for **DNT**, in contrast to **DNO** where it was the dominant reaction.

**Figure 2:**
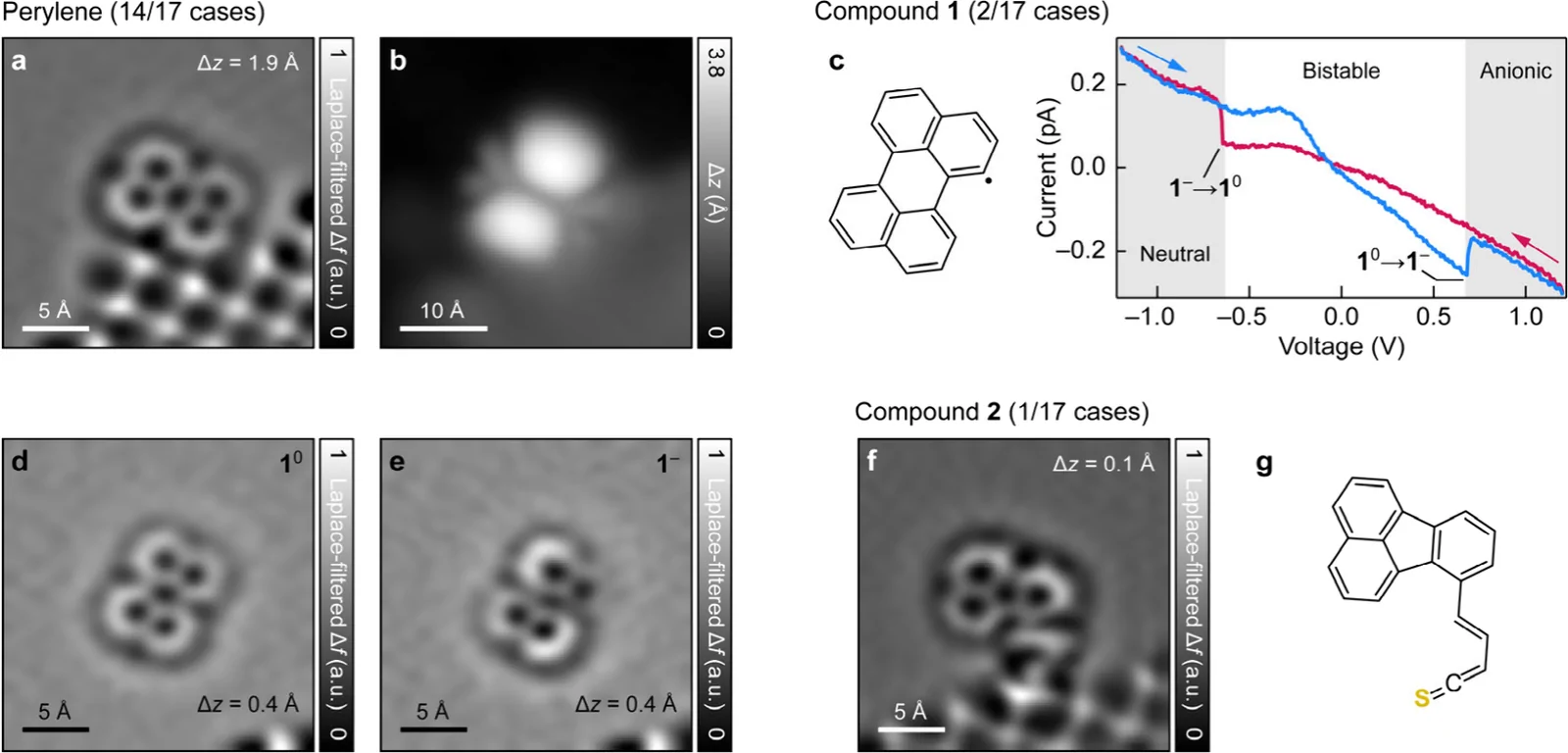
Structural and electronic characterization of reaction products on bilayer NaCl. (a) Laplace-filtered AFM image of a perylene molecule adsorbed at the step edge of a third-layer NaCl island. The bright and dark features in the third-layer NaCl island correspond to Cl^–^ and Na^+^ ions, respectively. (b) STM image of the perylene molecule in (a) showing its LUMO density (*V* = 2.0 V, *I* = 0.3 pA). (c) Constant-height *I*(*V*) spectra acquired on **1** (right), revealing charge-state bistability. Open feedback parameters: *V* = −1.2 V, *I* = 0.3 pA; forward (backward) sweeps: −1.2 to 1.2 V (1.2 V to −1.2 V). The molecule is in the neutral (anionic) state for *V* < −0.65 V (*V* > 0.68 V). The hysteretic behavior of the charging (**1** ^**0**^ → **1** ^**–**^) and discharging (**1** ^**–**^ → **1** ^**0**^) processes is related to the reorganization energy.^17,18^ Within the hysteresis loop, **1** is bistable and can be imaged in both charge states. Also shown is the chemical structure of **1** (left). (d, e) Laplace-filtered AFM images of **1** ^**0**^ (left) and **1** ^**–**^ (right). (f, g) Laplace-filtered AFM image of **2** adsorbed at the step edge of a third-layer NaCl island (left) and the proposed chemical structure of **2** (right). STM set-point for AFM images: *V* = 0.2 V and *I* = 0.5 pA on bilayer NaCl. The images in (d) and (e) were acquired with the same tip, while those in (a), (b), and (f) were acquired with different tips.

Figure 2a shows an AFM image of a perylene molecule generated via tip-induced removal of the sulfur atom from **DNT** (see also Figure S9). STM imaging of the molecule at *V* = 2 V revealed the lowest unoccupied molecular orbital (LUMO) density (Figure 2b), which agreed well with the calculated LUMO of perylene (Figure S10). This reinforced the assignment of the molecule as perylene. In a minority of cases, we also observed the generation of compound **1**, which exhibited charge-state bistability between neutral (denoted **1** ^**0**^) and anionic (denoted **1** ^**–**^) states (Figure 2c). AFM imaging revealed that whereas **1** ^**0**^ is planar (Figure 2d), the trivalent carbon atom in **1** ^**–**^ binds to the surface, leading to a nonplanar adsorption geometry of **1** ^**–**^ (Figure 2e). The assignment of charge states of **1** is supported by the absence (presence) of NaCl/Cu(111) interface-state scattering^19,20^ by the neutral (charged) species (Figure S11). Finally, in one case, we observed the generation of compound **2** resulting from a ring-opening and cyclization side reaction. Figure 2f,g shows the AFM image and chemical structure of **2**, which consists of a fluoranthenyl core along with a functional group that we tentatively assign as buta-1,3-diene-1-thione.

To elucidate the key experimental observations, namely, the predominance of atom deletion and the absence of ring-contraction rearrangement in skeletal editing of **DNT** relative to **DNO**, we performed DFT calculations of the reaction landscape of **DNT** and compared the results with our previous findings for **DNO**.^16^ Figure 3a,b shows the reaction paths and energy barriers toward the generation of perylene and **P1** in the gas phase. See Figure S12 for calculations on bilayer NaCl/Cu(111). Perylene results from atom deletion and is the predominant product of tip-induced reactions of **DNT**. **P1**, which results from ring-contraction rearrangement, was not observed in the present study but was the major product of tip-induced reactions of **DNO**. Starting from **DNT** (Figure 3a), the first step involves migration of the sulfur atom to a C–C bridge site and formation of a central six-membered ring. The formation of the intermediate **Int1­(S)** is exothermic and associated with an activation energy Δ*E* ^⧧^ = 1.70 eV (the corresponding reaction **DNO** → **Int1­(O)** is endothermic and has Δ*E* ^⧧^ = 2.70 eV; see Figure 3b). From **Int1­(S)**, three reaction paths are possible, as for the case of **Int1­(O)**. First, the sulfur atom can be directly eliminated, resulting in the generation of perylene. The associated Δ*E* ^⧧^ for **Int1­(S)** → perylene is 2.08 eV, substantially lower than Δ*E* ^⧧^ = 3.85 eV for **Int1­(O)** → perylene. Second, the sulfur atom can migrate to a neighboring bridge site, resulting in the intermediate **Int2­(S)** (Δ*E* ^⧧^ = 0.54 eV), from where it can again be eliminated to generate perylene, associated with Δ*E* ^⧧^ = 1.80 eV (for **Int2­(O)** → perylene, Δ*E* ^⧧^ = 3.44 eV). Third, a rearrangement reaction **Int1** → **Int3** can occur, which exhibits similar Δ*E* ^⧧^ values of 1.03 and 1.20 eV for the sulfur and oxygen cases, respectively, and is exothermic. From **Int3**, removal of the hydrogen atom bonded to the chalcogen results in the generation of the ring-contraction-rearrangement product **P1**, which exhibits similar Δ*E* ^⧧^ values of 3.16 and 3.28 eV for the sulfur and oxygen cases, respectively. The substantially lowered Δ*E* ^⧧^ (by more than 1.60 eV) for chalcogen elimination from **Int1** and **Int2** for sulfur compared to oxygen, along with the similar Δ*E* ^⧧^ values for the reaction **Int3** → **P1** for sulfur and oxygen (the difference being only 0.12 eV), results in the high selectivity for atom deletion in **DNT**.

**Figure 3:**
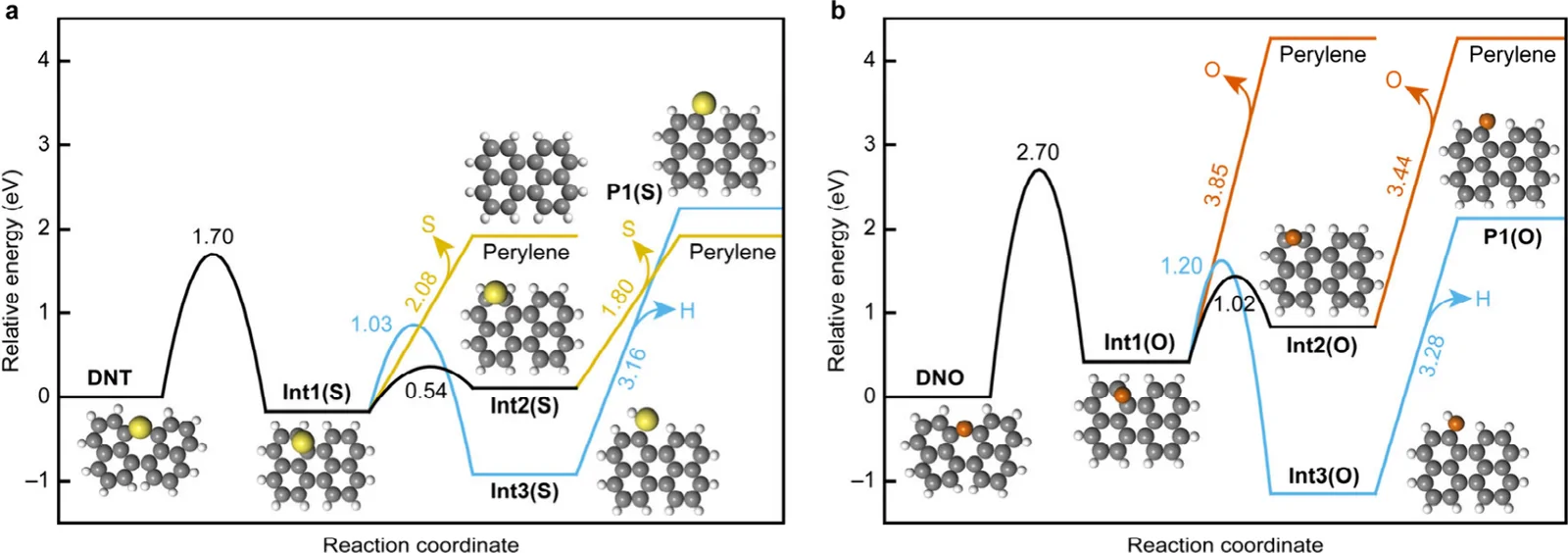
Gas-phase potential energy landscapes of skeletal editing of (a) **DNT** and (b) **DNO** obtained by DFT calculations. The numbers denote activation energies of each reaction. For **Int1** and **Int2** → perylene and **Int3** → **P1**, energy differences coincide with activation energies. Also shown are the optimized molecular geometries of precursors, intermediates, and products. Atomic color codes are the same as in Figure 1, with O shown in orange. The calculations were performed assuming a neutral charge state of the reactant, intermediates, and products. Panel (b) is adapted from ref 16. CC BY 4.0.

In conclusion, we demonstrate selectivity in skeletal editing of a heterocyclic core by the choice of the heteroatom. Combining single-molecule scanning probe experiments and density functional theory calculations, we show that tip-induced reactions of dinaphtho­[1,8-*bc*:1′,8′-*ef*]­thiepine adsorbed on NaCl predominantly lead to atom deletion. This is in contrast to our previous study on the oxygen-containing analogue dinaphtho­[1,8-*bc*:1′,8′-*ef*]­oxepine, where both ring-contraction rearrangement and atom deletion were observed as the majority and minority reactions, respectively. Our results demonstrate selective skeletal editing for a specific molecular system, and future directions could thus involve exploring the influence of molecular geometry, insulating surface, and molecular charge state on the reaction selectivity. Establishing control over atom deletion and ring-contraction rearrangement pathways could enable diverse applications in on-surface chemistry, such as the synthesis of elusive molecules (polyradicals or molecules with strained rings) by atom extrusion or imprinting electronic functionalities in graphene nanoribbons by edge functionalization.^21,22^

## Supplementary Material



## Notes

Experimental and theoretical methods, solution-phase synthesis of **DNT**, NMR characterization of **DNT**, STM and AFM data, and DFT calculations of molecules adsorbed on bilayer NaCl/Cu(111) (PDF)
