# Supplementary material for: Selectivity in Tip-Induced Skeletal Editing via Heteroatom Substitution
Source: J Am Chem Soc. 2026 Sep 24;148(39):41755–9. doi: 10.1021/jacs.6c12567 (PMC13651514; doi:10.1021/jacs.6c12567)
Supplement: Supplementary file 1 [file ja6c12567_si_001.pdf]

Supporting Information

Selectivity in tip-induced skeletal editing via heteroatom substitution

Shantanu Mishra,[1] Rasmus Svensson,[1,2] Valentina Malave,[3] Florian Albrecht,[4] Manuel Vilas-Varela,[3] Henrik Grönbeck,[1,2] Leo Gross[4] and Diego Peña[3,5]

[1]Department of Physics and Astronomy, Chalmers University of Technology, 412 96 Göteborg, Sweden

[2]Competence Centre for Catalysis, Chalmers University of Technology, 412 96 Göteborg, Sweden

[3]Center for Research in Biological Chemistry and Molecular Materials, and Department of Organic Chemistry, University of Santiago de Compostela, 15782 Santiago de Compostela, Spain

[4]IBM Research, 8803 Rüschlikon, Switzerland

[5]Oportunius, Galician Innovation Agency, 15702 Santiago de Compostela, Spain

Contents:

1. Methods Page S2
2. Scanning probe data and calculations Page S5
3. References Page S12

1. Methods

1.1. Solution-phase synthesis and characterization

Starting materials were purchased reagent grade from TCI and Sigma-Aldrich and used without further purification. Binaphthalene **3** was synthesized following a reported procedure.[1] All reactions were performed in oven-dried glassware under an inert atmosphere of purified argon using Schlenk techniques. Thin-layer chromatography was performed on Silica Gel 60 F-254 plates (Merck). Column chromatography was performed on silica gel (40-60 µm). Nuclear magnetic resonance (NMR) spectra were recorded on a Varian Inova 300 spectrometer.

Scheme S1. Synthesis of DNT.

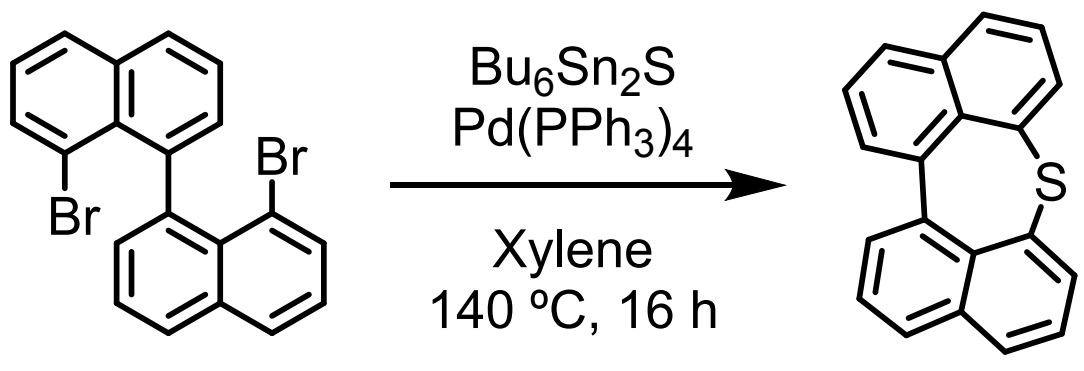

Over a degassed mixture of **3** (500 mg, 1.21 mmol) and Bu_6Sn_2S (630 µL, 1.21 mmol) in xylene (40 mL), the catalyst $Pd(PPh_3)_4$ (138 mg, 0.10 mmol) was added (Scheme S1). The resulting mixture was stirred at 140 ºC for 16 h. Then, the solvent was evaporated under reduced pressure, and the remaining residue was purified by column chromatography (SiO_2; hexane:CH_2Cl_2 9:1), affording **DNT** (14 mg, 4%) as a yellow solid.

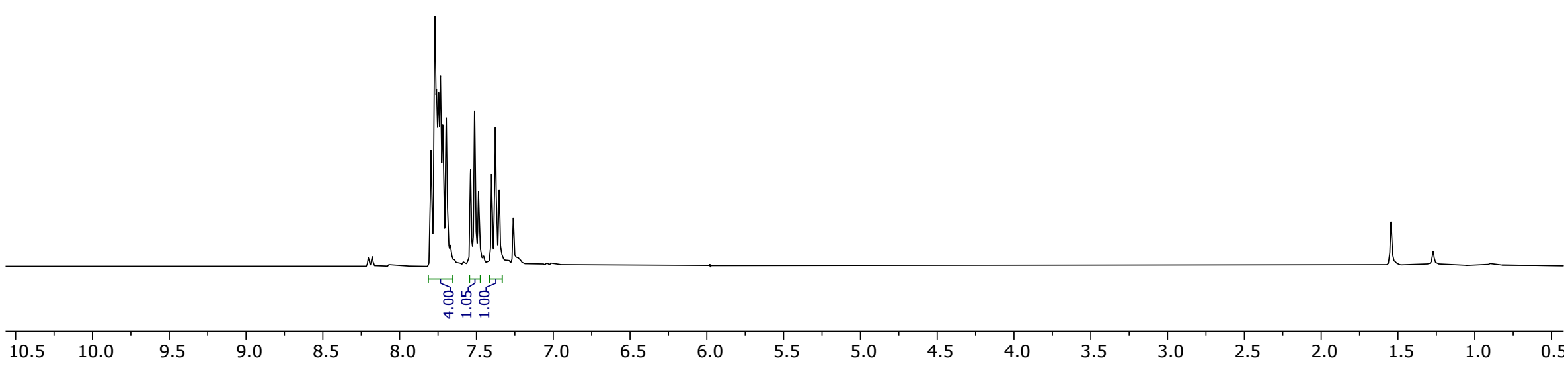

Figure S1. ^{1}H NMR spectrum of **DNT**.

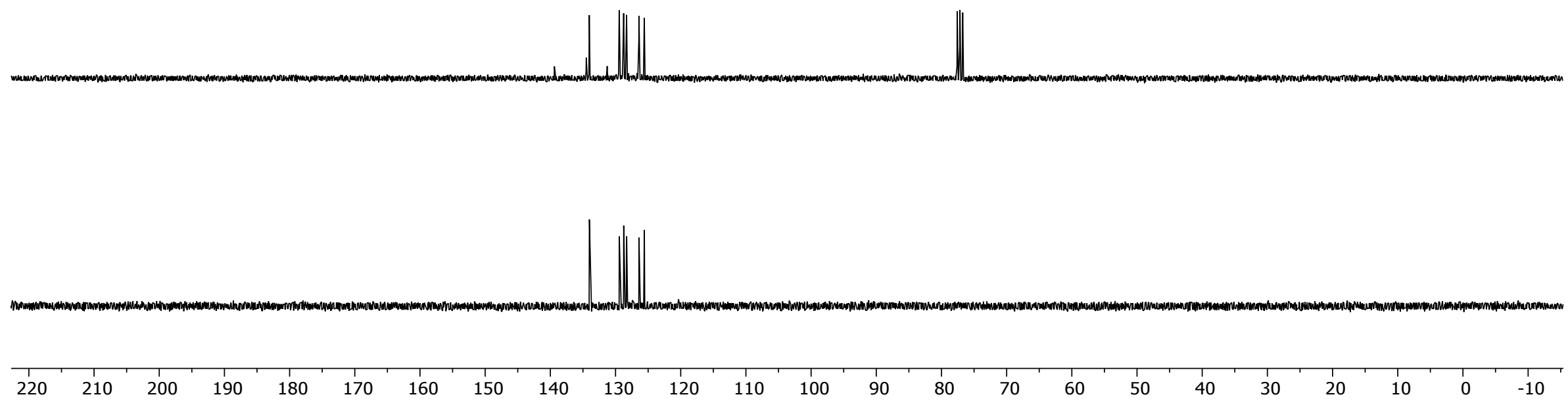

Figure S2. ^{13}C NMR-DEPT spectra of **DNT** (DEPT: Distortionless Enhancement by Polarization Transfer).

1.2. Sample preparation and scanning probe microscopy measurements

STM and AFM measurements were performed in a home-built system operating at base pressures below 1×10^{-10} mbar and a base temperature of 5 K. Bias voltages were applied to the sample with respect to the tip. Unless noted otherwise, all measurements (including application of voltage pulses) were performed with copper-coated PtIr tips functionalized with a single carbon monoxide molecule at the tip apex. AFM imaging was performed in non-contact mode using a qPlus sensor.[2] The sensor was operated in frequency-modulation mode[3] with a constant oscillation amplitude of 0.5 Å. STM imaging was performed in constant-current mode, and AFM imaging was performed in constant-height mode at 0 V. STM and AFM images were post-processed using Gaussian low-pass filters.

The Cu(111) surface was prepared by multiple cycles of sputtering with Ne^+ ions and annealing up to 823 K. NaCl was thermally evaporated onto the Cu(111) surface held at 284 K. This protocol resulted in the growth of large and defect-free bilayer (100)-terminated NaCl films, with a minority of third-layer NaCl islands. The sample quality was checked by STM imaging prior to further preparation. Submonolayer coverage of **DNT** molecules on the surface was obtained by flashing an oxidized silicon wafer containing the molecules in front of the cold sample in the microscope. Carbon monoxide molecules were dosed from the gas phase onto the cold sample. The maximum sample temperature reached during deposition of **DNT** and carbon monoxide molecules was 16 K.

1.3. Density functional theory calculations

Density functional theory calculations were performed using the Vienna Ab Initio Simulation Package (VASP).[4–7] Interactions between core and valence electrons were described using the frozen core projector augmented wave (PAW) method.[8,9] The Kohn-Sham orbitals were expanded with plane waves, truncated at an energy cutoff of 450 eV. The considered valence electrons were $1s^1$ (H), $2s^2 2p^2$ (C), $2p^6 3s^1$ (Na), $3s^2 3p^4$ (S), $3s^2 3p^5$ (Cl) and $4s^1 3d^{10}$ (Cu). Exchange and correlation effects were described with the Perdew-Burke-Ernzerhof (PBE) functional.[10] For surface adsorptions, Grimme's D3 correction was included to account for the

dispersion interactions.[11,12] The electronic structure was optimized until changes in the electronic energy and Kohn-Sham eigenvalues were below 1×10^{-6} eV between succeeding iterations, whereas the nuclear structure was regarded as converged when the maximum nuclear force was below 0.03 eV/Å.

The lattice constant of NaCl was determined using a $p(1 \times 1)$ bilayer of NaCl (consisting of 4 Na and 4 Cl atoms), and optimized to be 5.53 Å. The lattice constant of bulk Cu was determined to be 3.57 Å, slightly lower than the experimental value of 3.61 Å.[13] The gas-phase species were optimized using a (32, 31, 30) Å vacuum box. Transition state calculations were performed using the climbing-image nudged elastic band method[14,15] using a (19, 18, 15) Å vacuum box. Molecular orbitals of the gas-phase molecules were calculated using Dmol^3 (ref.[16]). The Brillouin zones for all calculations were sampled by the Γ-point. The adsorption configuration of **DNT** was determined on a bilayer of $(5\sqrt{2} \times 4\sqrt{2})$R45° NaCl(100), supported on 2 layers of $(11 \times 5\sqrt{3})$rect. Cu(111). The optimized lattice constant for NaCl was used, whereas the Cu surface was strained to match the lattice of bilayer NaCl. The strains on Cu(111) were –0.50% and 1.11% in the two dimensions, respectively. In the calculations, the bottom layer of the strained Cu(111) was kept fixed, so as to emulate a bulk system.

2. Scanning probe data and calculations

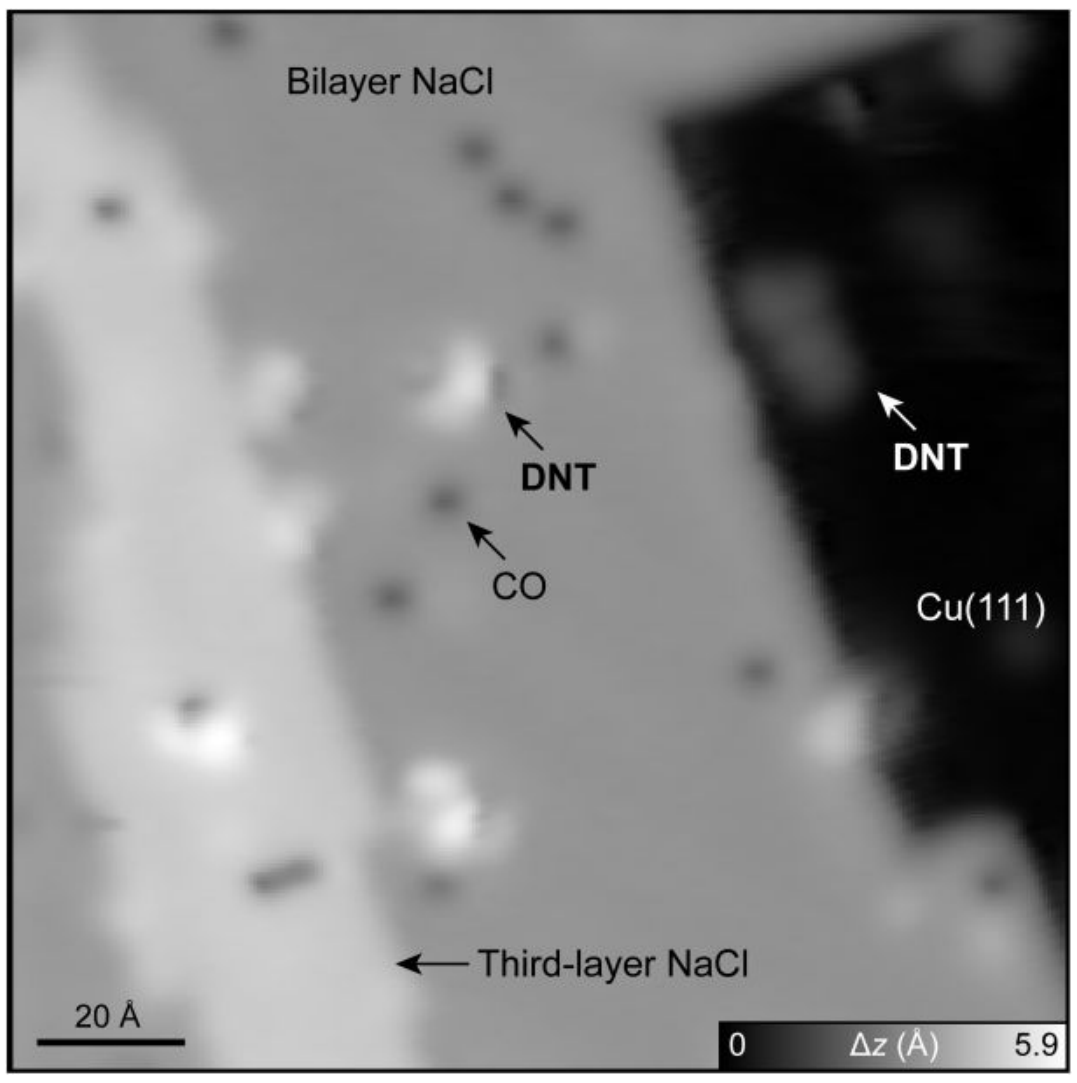

Figure S3. STM overview image of the sample. Scanning parameters: $V = 0.7$ V and $I = 0.3$ pA. Individual **DNT** molecules on NaCl and Cu(111), and co-adsorbed carbon monoxide (CO) molecules on NaCl are labeled. The image was acquired with a copper-coated metallic tip.

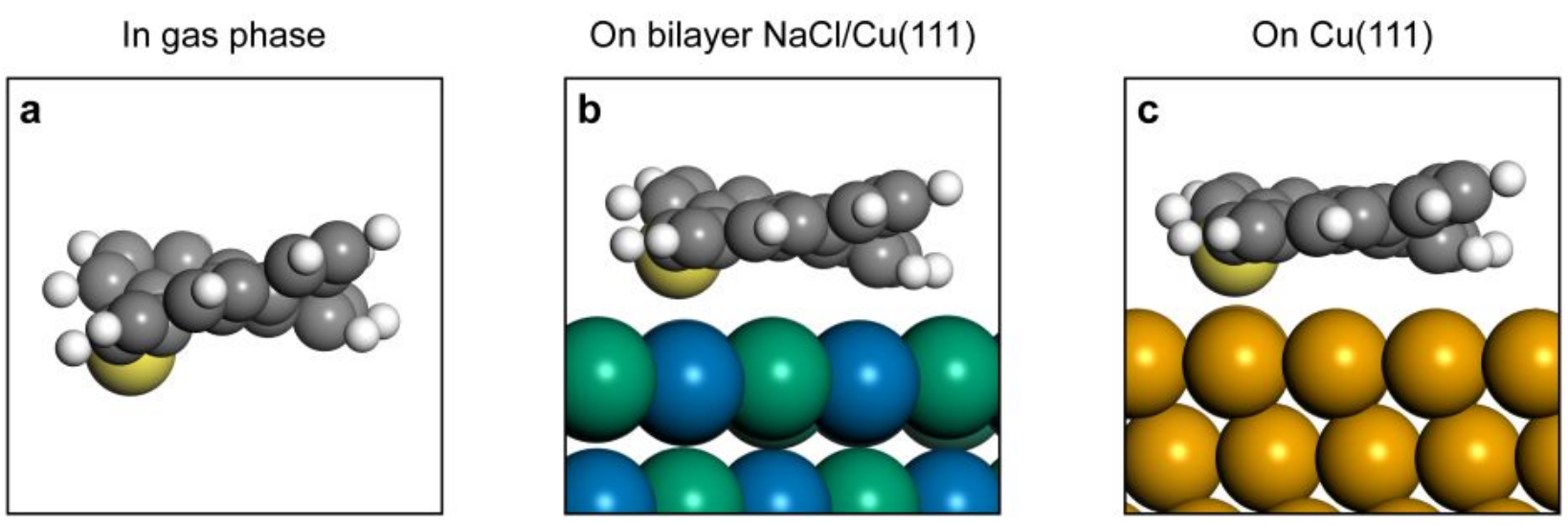

Figure S4. Side views of the DFT-calculated geometries of **DNT** in gas phase (a) and adsorbed on bilayer NaCl/Cu(111) (b) and Cu(111) (c). The progressive planarization of **DNT** from (a) to (c) is due to increasing molecule-surface interaction.

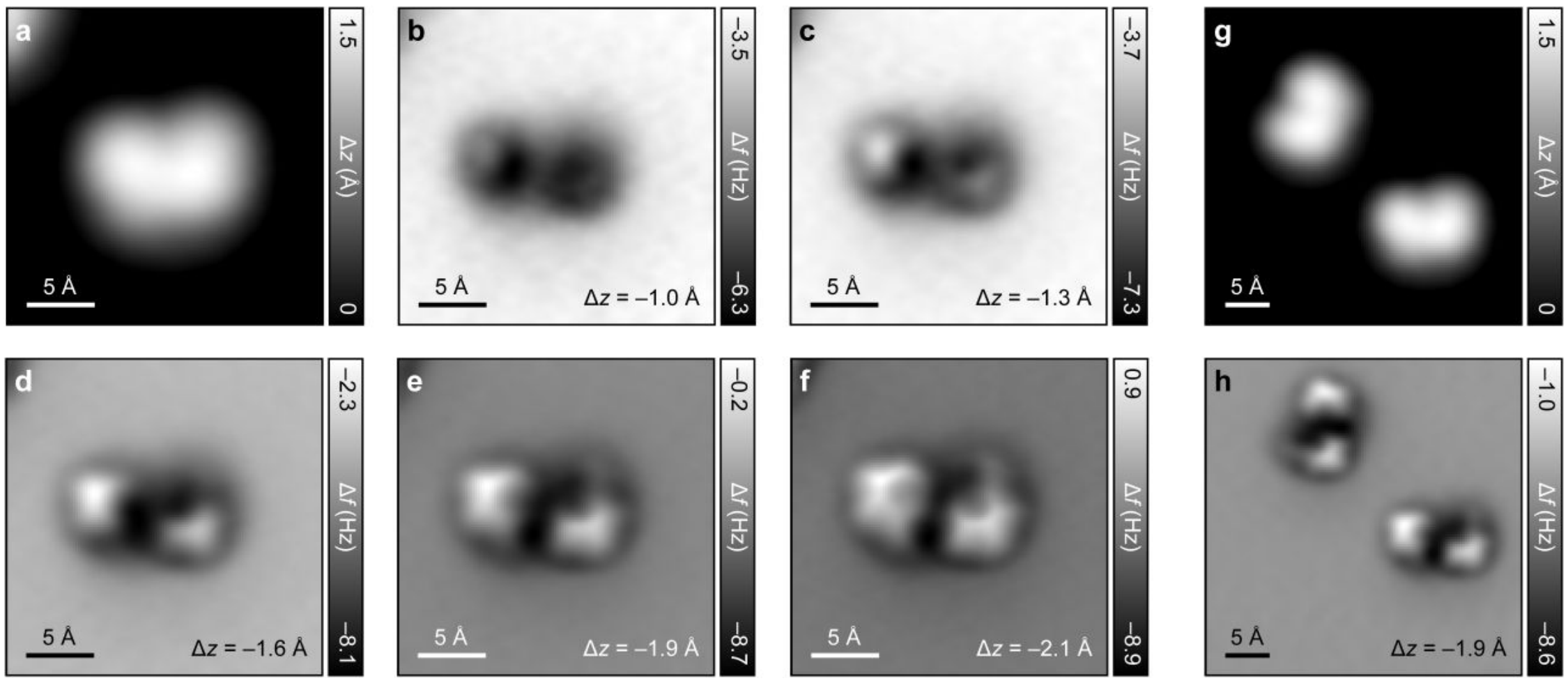

Figure S5. (a) STM image of a **DNT** molecule on Cu(111). (b–f) AFM images of the **DNT** molecule shown in (a) at different Δ*z* values. (g, h) STM (g) and AFM (h) images of two atropisomers of **DNT** on Cu(111). Scanning parameters for the STM image: *V* = 0.2 V and *I* = 0.3 pA. STM set-point for AFM images: *V* = 0.2 V and *I* = 0.5 pA on Cu(111). All images were acquired with the same tip.

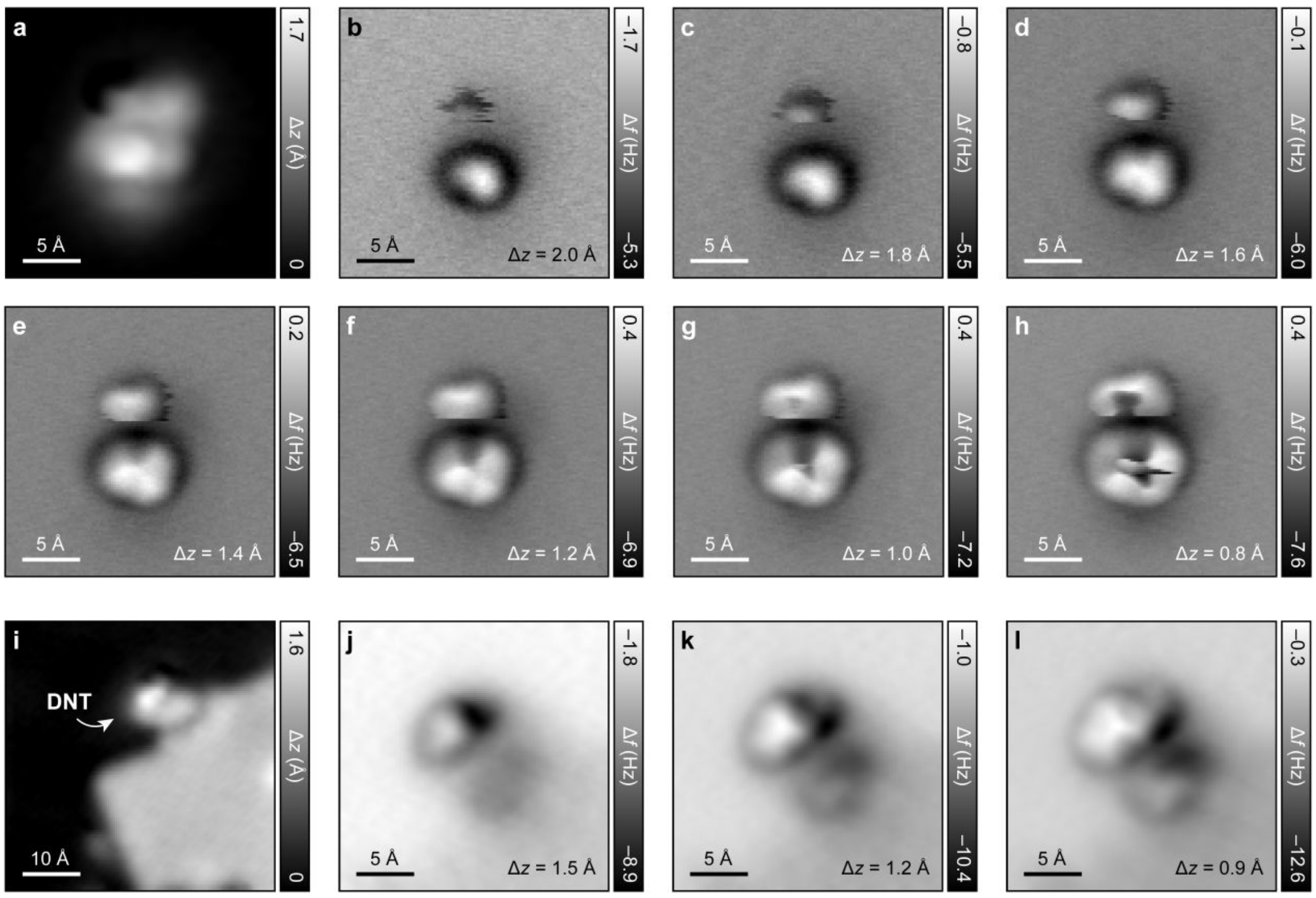

Figure S6. (a) STM image of a **DNT** molecule on bilayer NaCl. (b–h) AFM images of the **DNT** molecule shown in (a) at different Δ*z* values. (i) STM image of a **DNT** molecule adsorbed at the step edge of a third-layer NaCl island. (j–l) AFM images of the **DNT** molecule shown in (i) at different Δ*z* values. The adsorption of the molecule is stabilized at the step edge and conformational switching is suppressed, evidenced by the absence of streaks in AFM imaging. Scanning parameters for STM images: *V* = 0.2 V, and *I* = 0.25 pA (a) and *I* = 0.50 pA (i). STM

set-point for AFM images: $V = 0.2$ V, and $I = 0.25$ pA (b–h) and $I = 0.50$ pA on NaCl. The images in (a–h) were acquired with the same tip, whereas those in (i–l) were acquired with a different tip.

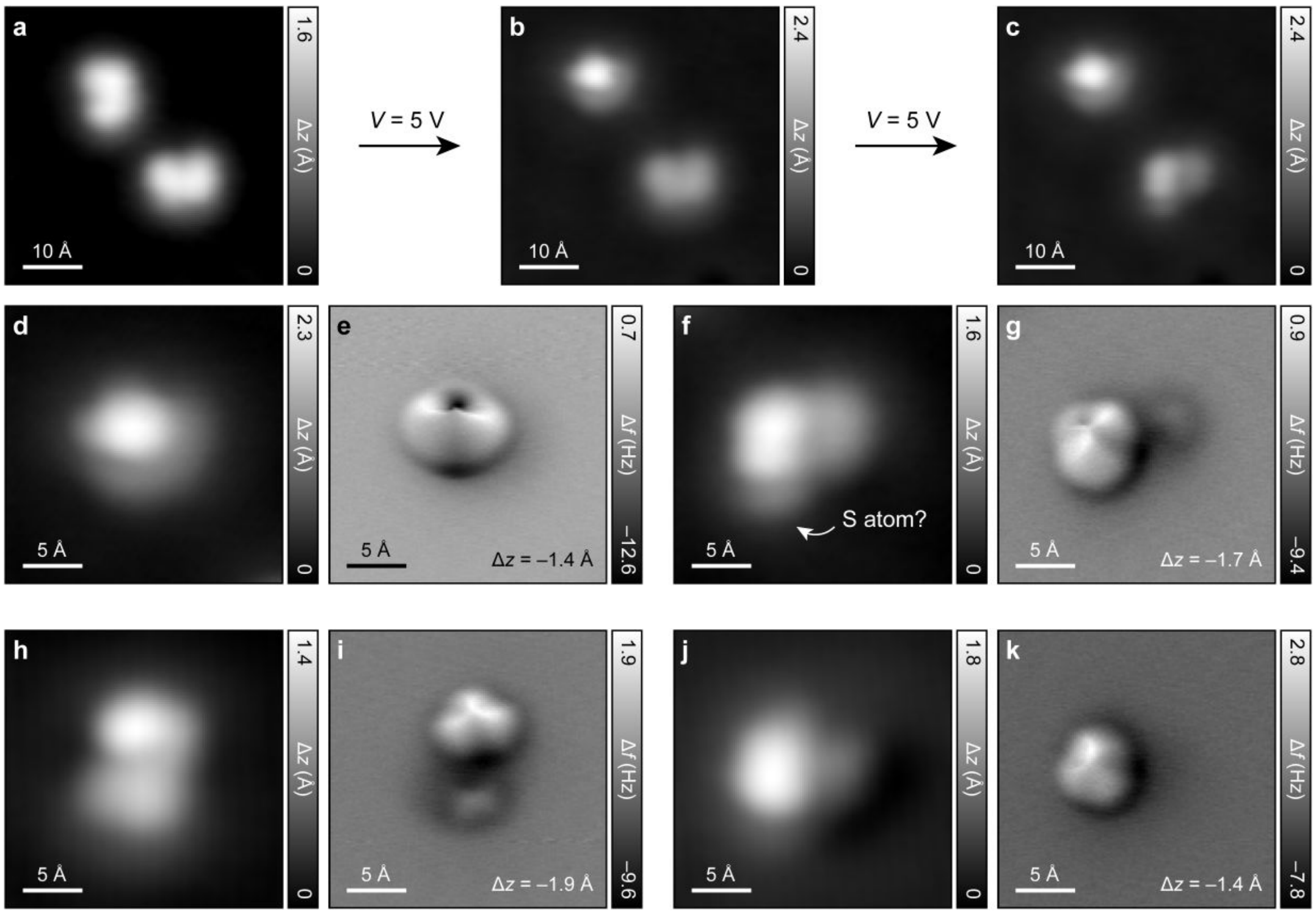

Figure S7. (a–c) STM images showing sequential tip-induced manipulation of **DNT** molecules on Cu(111): starting from two **DNT** molecules in (a), a voltage pulse of 5.0 V was first applied to the top molecule (b) and subsequently to the bottom molecule (c). (d–g) STM (d, f) and corresponding AFM (e, g) images of the molecules in (c). The species in (d, e) is difficult to identify. For the species in (f, g), two benzenoid rings (one of which exhibits a strong upward tilt) can be identified in (g). It is likely that the sulfur atom was abstracted from the molecule, resulting in the generation of a σ radical. The indicated feature in (f) could correspond to the detached sulfur atom. The strong non-planarity of the molecule probably results from the formation of C–Cu bond at the radical position(s). (h–k) STM (h, j) and AFM (i, k) images of two other species generated via application of voltage pulses of 5.0 V (h, i) and 5.1 V (j, k) to **DNT** molecules on Cu(111). The species in (h, i) and (j, k) exhibit different degrees of non-planarity. It is unclear from the images if the voltage pulses also triggered cleavage of C(sp^2)–H bonds. Perylene molecules were not obtained on Cu(111). Scanning parameters for STM images: $V = 0.2$ V and $I = 0.3$ pA. STM set-point for AFM images: $V = 0.2$ V and $I = 0.5$ pA on NaCl. All images were acquired with the same tip.

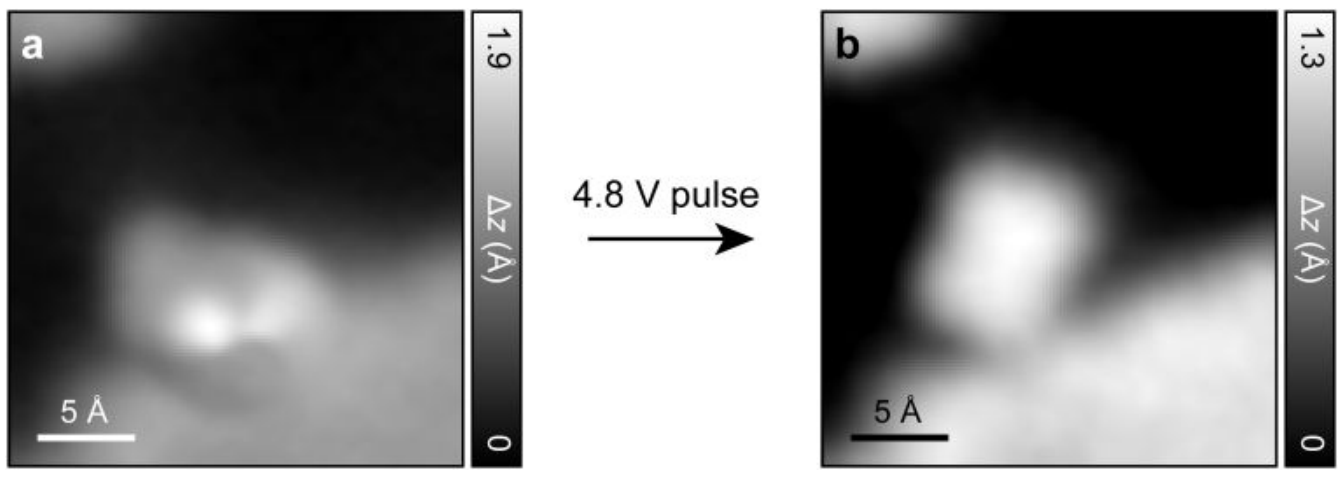

Figure S8. (a) STM image of a **DNT** molecule adsorbed on bilayer NaCl at the step edge of a third-layer NaCl island. (b) STM image of the same area after application of a voltage pulse of 4.8 V to the molecule. The species in (b) corresponds to a perylene molecule, whose AFM image is shown in Figure S9a. Scanning parameters for STM images: V = 0.2 V and I = 0.3 pA. Before ramping the voltage to 4.8 V, the tip was retracted by Δz = 10 Å from the STM set-point of V = 0.2 V and I = 0.5 pA on top of the **DNT** molecule.

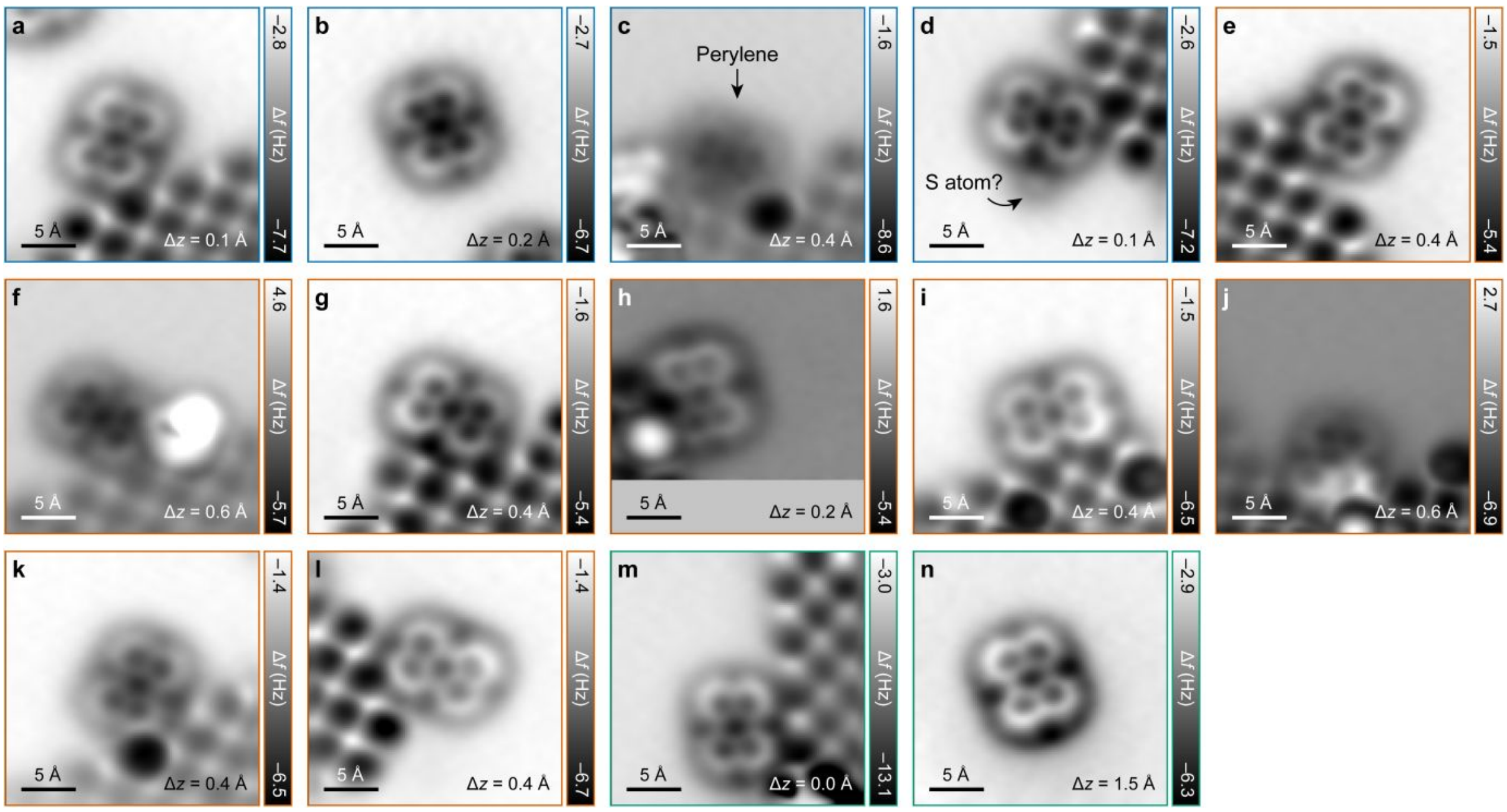

Figure S9. AFM images of all 14 perylene molecules generated via application of voltage pulses to **DNT** molecules on bilayer NaCl. The adsorbate indicated in (d) could correspond to a detached sulfur atom. This adsorbate was found to be negatively charged on the surface, inferred through the observation of NaCl/Cu(111) interface-state scattering by the adsorbate. The molecule in (j) is adsorbed at a copper step edge overgrown by NaCl. The molecule in (n) is adsorbed on a third-layer NaCl island. Images with the same frame colors were acquired with the same tip. STM set-point for AFM images: V = 0.2 V and I = 0.5 pA on NaCl.

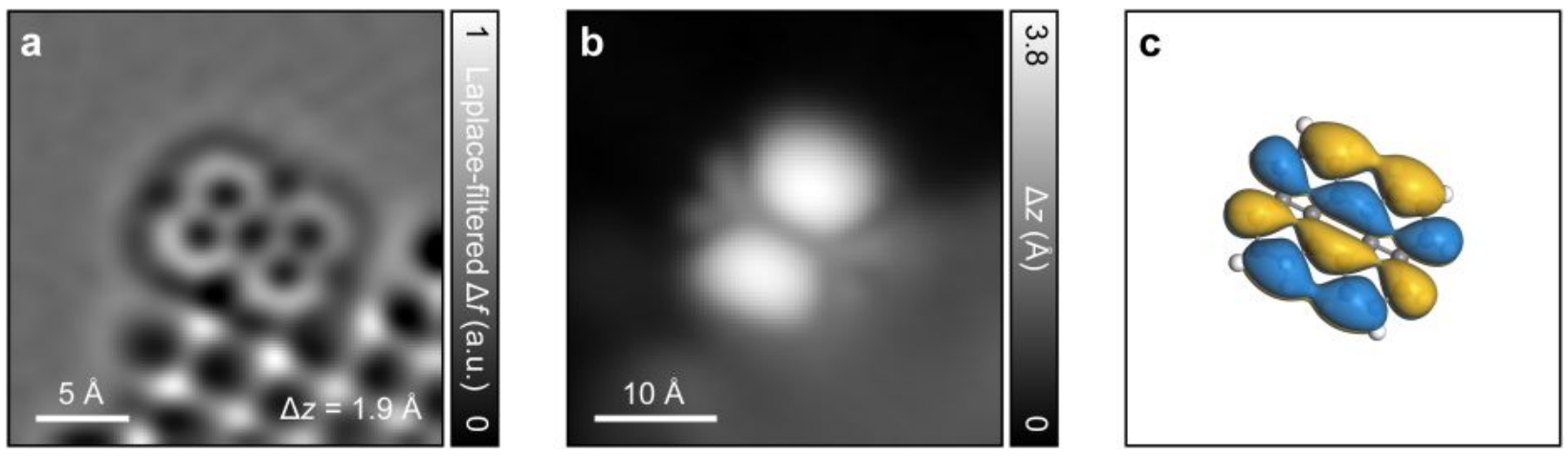

Figure S10. (a) Laplace-filtered AFM image of a perylene molecule generated on bilayer NaCl. (b) STM image of the perylene molecule in (a) showing its LUMO density (V = 2 V, I = 0.3 pA). Images (a) and (b) are also shown in Figure 2 of the main text. (c) The DFT-calculated LUMO of perylene (isosurface: $0.01a_0^{-3/2}$, a_0 denotes the Bohr radius).

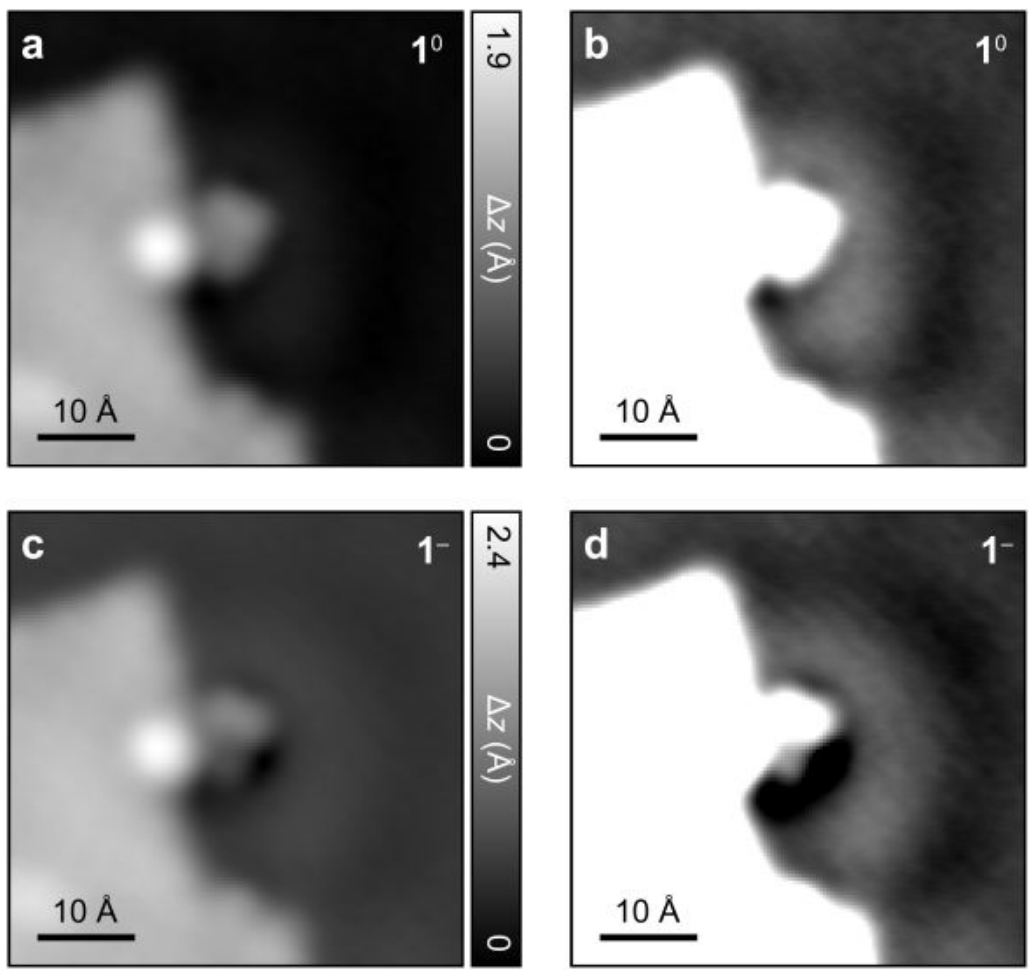

Figure S11. STM images of **1**, adsorbed on bilayer NaCl at the step edge of a third-layer NaCl, in the neutral (a, b) and anionic (c, d) states. Image of **1** in each charge state is shown with two contrast levels. Strong NaCl/Cu(111) interface-state scattering[17,18] by **1**⁻ is observed (d), supporting its charged state. Scanning parameters: V = 0.2 V and I = 0.3 pA. The voltage in (a–d) is chosen to lie above the onset of NaCl/Cu(111) interface state at V ~ –0.2 V.[17]

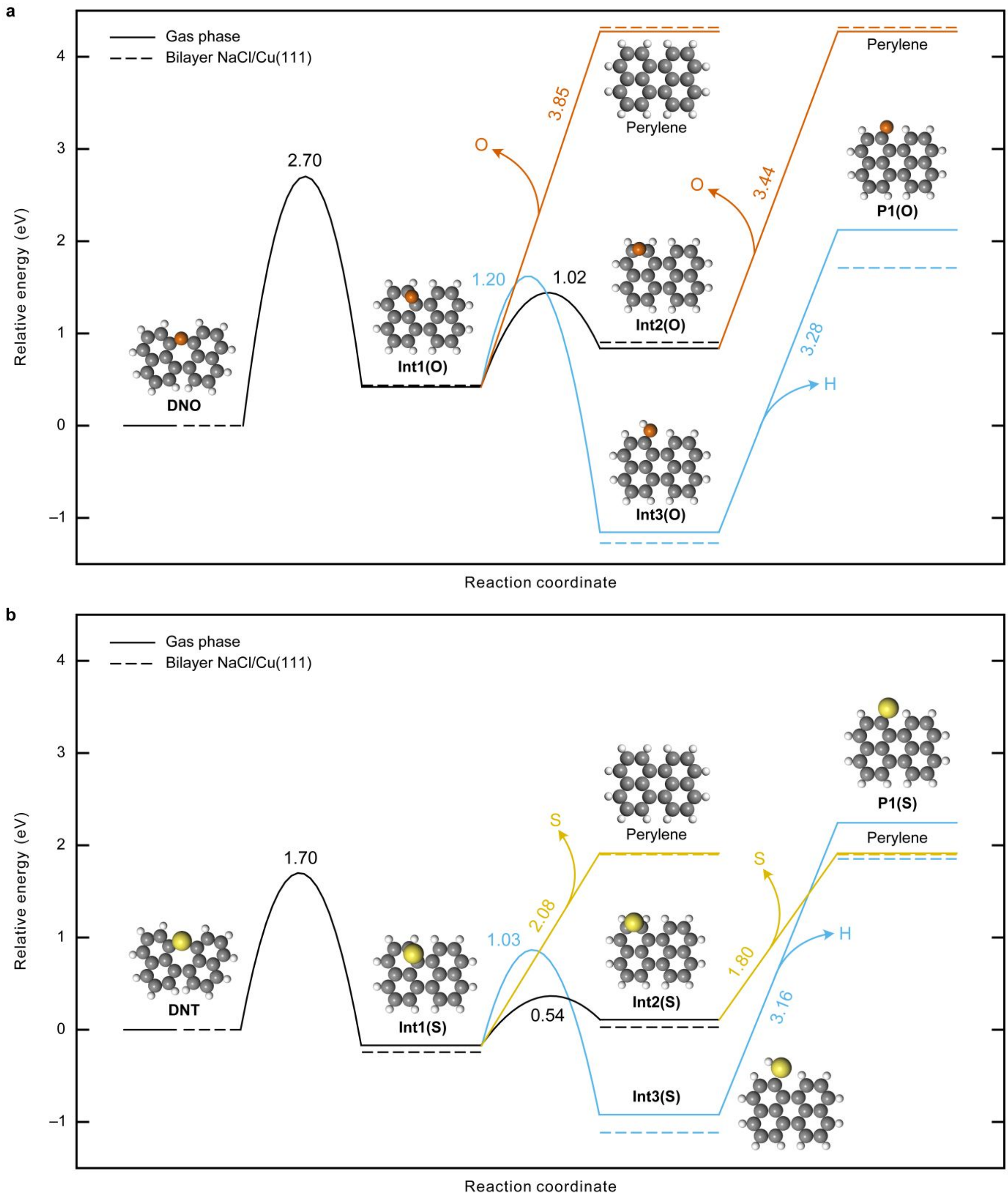

Figure S12. DFT calculations of the potential energy landscapes in the gas phase (solid lines) and on bilayer NaCl/Cu(111) (dashed lines) for skeletal editing of **DNO** (a) and **DNT** (b). The gas-phase calculations, also shown in Figure 3 in the main text, reveal a substantial difference in the reaction landscapes of **DNT** and **DNO**. Replacing oxygen with sulfur leads to a reduction (by more than 1.60 eV) in the activation energies for chalcogen elimination from **Int1** and **Int2** to form perylene, whereas the barrier for **Int3** → **P1** is similar for the sulfur and oxygen cases (the difference in activation energies for the two cases being only 0.12 eV). This is the single most important reason for the observed selectivity for atom deletion in **DNT**. The calculated potential energy landscapes on bilayer NaCl/Cu(111) follow the same trend for **DNT** and

DNO. The relative energies of the intermediates **Int1**–**Int3** on bilayer NaCl/Cu(111) are similar to those in the gas phase for the oxygen and sulfur cases, whereas **P1** is stabilized on bilayer NaCl/Cu(111) by roughly the same amount for the oxygen and sulfur cases. The overall picture on bilayer NaCl/Cu(111) thus remains the same as that obtained from the gas-phase calculations; that is, the barrier for atom deletion relative to ring-contraction rearrangement is strongly reduced for **DNT** compared to **DNO**, which results in the observed selectivity for atom deletion in **DNT**. Panel (a) is adapted from ref.[19]. Copyright 2025 American Chemical Society.

3. References

(1) Regulska, E.; Ruppert, H.; Rominger, F.; Romero-Nieto, C. Synthesis of Blue-Luminescent Seven-Membered Phosphorus Heterocycles. *J. Org. Chem.* **2020**, *85* (2), 1247–1252.

(2) Giessibl, F. J. High-Speed Force Sensor for Force Microscopy and Profilometry Utilizing a Quartz Tuning Fork. *Appl. Phys. Lett.* **1998**, *73* (26), 3956–3958.

(3) Albrecht, T. R.; Grütter, P.; Horne, D.; Rugar, D. Frequency Modulation Detection Using high-Q Cantilevers for Enhanced Force Microscope Sensitivity. *J. Appl. Phys.* **1991**, *69* (2), 668–673.

(4) Kresse, G.; Hafner, J. Ab Initio Molecular Dynamics for Liquid Metals. *Phys. Rev. B* **1993**, *47* (1), 558–561.

(5) Kresse, G.; Hafner, J. Ab Initio Molecular Dynamics for Open-Shell Transition Metals. *Phys. Rev. B* **1993**, *48* (17), 13115–13118.

(6) Kresse, G.; Hafner, J. Ab Initio Molecular-Dynamics Simulation of the Liquid-Metal–Amorphous-Semiconductor Transition in Germanium. *Phys. Rev. B* **1994**, *49* (20), 14251–14269.

(7) Kresse, G.; Furthmüller, J. Efficient Iterative Schemes for Ab Initio Total-Energy Calculations Using a Plane-Wave Basis Set. *Phys. Rev. B* **1996**, *54* (16), 11169–11186.

(8) Blöchl, P. E. Projector Augmented-Wave Method. *Phys. Rev. B* **1994**, *50* (24), 17953–17979.

(9) Kresse, G.; Joubert, D. From Ultrasoft Pseudopotentials to the Projector Augmented-Wave Method. *Phys. Rev. B* **1999**, *59* (3), 1758–1775.

(10) Perdew, J. P.; Burke, K.; Ernzerhof, M. Generalized Gradient Approximation Made Simple. *Phys. Rev. Lett.* **1996**, *77* (18), 3865–3868.

(11) Grimme, S.; Antony, J.; Ehrlich, S.; Krieg, H. A Consistent and Accurate Ab Initio Parametrization of Density Functional Dispersion Correction (DFT-D) for the 94 Elements H-Pu. *J. Chem. Phys.* **2010**, *132* (15), 154104.

(12) Grimme, S.; Ehrlich, S.; Goerigk, L. Effect of the Damping Function in Dispersion Corrected Density Functional Theory. *J. Comput. Chem.* **2011**, *32* (7), 1456–1465.

(13) Crystal Structures and Lattice Parameters of Allotropes of the Elements. In CRC Handbook of Chemistry and Physics; Rumble, J. R., Ed.; 104th ed.; CRC Press, Inc., 2023.

(14) Mills, G.; Jónsson, H.; Schenter, G. K. Reversible Work Transition State Theory: Application to Dissociative Adsorption of Hydrogen. *Surf. Sci.* **1995**, *324* (2), 305–337.

(15) Henkelman, G.; Uberuaga, B. P.; Jónsson, H. A Climbing Image Nudged Elastic Band Method for Finding Saddle Points and Minimum Energy Paths. *J. Chem. Phys.* **2000**, *113* (22), 9901–9904.

(16) Delley, B. From Molecules to Solids with the $DMol^3$ Approach. *J. Chem. Phys.* **2000**, *113* (18), 7756–7764.

(17) Repp, J.; Meyer, G.; Rieder, K.-H. Snell's Law for Surface Electrons: Refraction of an Electron Gas Imaged in Real Space. *Phys. Rev. Lett.* **2004**, *92* (3), 036803.

(18) Swart, I.; Sonnleitner, T.; Repp, J. Charge State Control of Molecules Reveals Modification of the Tunneling Barrier with Intramolecular Contrast. *Nano Lett.* **2011**, *11* (4), 1580–1584.

(19) Mishra, S.; Malave, V.; Svensson, R.; Grönbeck, H.; Albrecht, F.; Peña, D.; Gross, L. Skeletal Editing by Tip-Induced Chemistry. *J. Am. Chem. Soc.* **2025**, *147* (48), 44055–44059.
